# Changes in prostate cancer survival among insured patients in relation to USPSTF screening recommendations

**DOI:** 10.1186/s12894-022-01045-0

**Published:** 2022-06-25

**Authors:** Isaac E. Kim, Daniel D. Kim, Sinae Kim, Shuangge Ma, Thomas L. Jang, Eric A. Singer, Saum Ghodoussipour, Isaac Yi Kim

**Affiliations:** 1 Warren Alpert Medical School, Brown University, Providence, RI USA; 2 Department of Biostatistics and Epidemiology, Rutgers School of Public Health, The State University of New Jersey, Piscataway, NJ USA; 3 Department of Epidemiology and Public Health, Yale University, New Haven, CT USA; 4 Section of Urologic Oncology, Rutgers Cancer Institute of New Jersey and Division of Urology, Rutgers Robert Wood Johnson Medical School, Rutgers, The State University of New Jersey, New Brunswick, NJ USA; 5 Department of Urology, Yale School of Medicine, 789 Howard Avenue, Fitkin 307, New Haven, CT 06520 USA

**Keywords:** Prostate cancer, Insurance, Survival disparity, SEER

## Abstract

**Background:**

To investigate the effects of the U.S. Preventive Services Task Force’s (USPSTF) 2012 recommendation against prostate-specific antigen (PSA)-based screening for prostate cancer on survival disparities based on insurance status. Prior to the USPSTF’s 2012 screening recommendation, previous studies found that insured patients with prostate cancer had better outcomes than uninsured patients.

**Methods:**

Using the SEER 18 database, we examined prostate cancer-specific survival (PCSS) based on diagnostic time period and insurance status. Patients were designated as belonging to the pre-USPSTF era if diagnosed in 2010–2012 or post-USPSTF era if diagnosed in 2014–2016. PCSS was measured with the Kaplan–Meier method, while disparities were measured with the Cox proportional hazards model.

**Results:**

During the pre-USPSTF era, uninsured patients experienced worse PCSS compared to insured patients (adjusted HR 1.256, 95% CI 1.037–1.520, p = 0.020). This survival disparity was no longer observed during the post-USPSTF era as a result of decreased PCSS among insured patients combined with unchanged PCSS among uninsured patients (adjusted HR 0.946, 95% CI 0.642–1.394, p = 0.780).

**Conclusions:**

Although the underlying reasons are not clear, the USPSTF’s 2012 PSA screening recommendation may have hindered insured patients from being regularly screened for prostate cancer and selectively led to worse outcomes for insured patients without affecting the survival of uninsured patients.

## Introduction

Prostate cancer is the most commonly diagnosed cancer and the second most common cause of cancer death for men in the United States with an estimated 34,130 deaths per year [1]. Previous studies in the pre-2012 era have reported significant disparities in prostate cancer based on insurance status. For instance, one study found that compared to insured patients, uninsured patients with prostate cancer present with higher prostate-specific antigen (PSA) levels and are more likely to present with higher Gleason scores and more advanced clinical T stage [2]. Moreover, studies have reported that uninsured patients with prostate cancer suffer mortality rates almost twice as high as those of insured patients [3].

The potential benefits of prostate-specific antigen (PSA)-based screening to reduce mortality has been debated largely due to the contradicting results of two major randomized controlled trials. In contrast to the European Randomized Study of Screening for Prostate Cancer (ERSPC) that reported a survival benefit to screening, the United States Prostate, Lung, Colorectal, and Ovarian (PLCO) Cancer Screening Trial did not find such a survival benefit. Moreover, the benefits of prostatectomy for PSA screen-detected prostate cancer remain unknown, because while the Scandinavian Prostate Cancer Group Study Number Four (SPCG-4) reported a survival benefit for prostatectomy, the Prostate Cancer Intervention Versus Observation Trial (PIVOT) did not [4].

Thus, the United States Preventive Services Taskforce (USPSTF) recommended against PSA-based screening for prostate cancer in May 2012 due to the aforementioned clinical trials and studies that reported that screening yielded few benefits in mortality and many potential harms such as complications from biopsies and subsequent treatment as well as the risk of overdiagnosis and overtreatment [5]. This recommendation prompted the American Urologic Association (AUA) and the Society of Urologic Oncology to issue a statement suggesting that the new recommendation would prevent early diagnosis and proper treatment of prostate cancer and fail to prevent otherwise avoidable cancer deaths [6]. Following the recommendation, several studies reported decreases in low-grade prostate cancer and increases in intermediate and high-risk cancer [7–9].

While previous studies have examined the effects of the USPSTF’s screening recommendation on racial disparities [10], to our knowledge, there have been no studies on the recommendation’s effect on survival according to insurance status. Because racial disparities in prostate cancer survival outcomes have been associated with socioeconomic factors, we hypothesized that the screening recommendation would result in the abrogation of survival disparities based on insurance status by discouraging prostate cancer screening.

## Materials and methods

### Data sources

This study examined patients who were at least 40-years-old and diagnosed with prostate cancer from the Surveillance, Epidemiology, and End Results (SEER) 18 registries census tract-level socioeconomic status database submitted in November 2018. This database contains patient information from the Alaska Native Tumor Registry, Connecticut, Detroit, Atlanta, Greater Georgia, Rural Georgia, San Francisco-Oakland, San Jose-Monterey, Greater California, Hawaii, Iowa, Kentucky, Los Angeles, Louisiana, New Mexico, New Jersey, Seattle-Puget Sound, and Utah.

### Study variables

Sociodemographic, clinicopathology, and treatment-related variables evaluated include insurance status, age, race, PSA, biopsy Gleason score (bGS), summary stage, and treatment with prostatectomy or radiotherapy. The SEER-designated summary stage incorporates both clinical and pathological information.

Three insurance categories were studied: insured, uninsured, and Medicaid. Given that Medicaid provides coverage for low-income individuals or those with disabilities, Medicaid and insured patients were treated as distinct groups to isolate potential confounding socioeconomic effects of Medicaid’s eligibility criteria on PCSS.

### Statistical analysis

Given our rationale that the screening recommendation would result in more advanced prostate cancer presentation and thus increased prostate cancer-specific mortality, the primary study outcome was prostate cancer-specific survival (PCSS) based on diagnostic time period and insurance status. Patients were designated as belonging to the pre-USPSTF era if diagnosed in 2010 to 2012 or post-USPSTF era if diagnosed in 2014 to 2016. 2013 was considered the buffer year to account for the time it would take for the new USPSTF recommendations to take effect.

PCSS was measured with the Kaplan–Meier method, while disparities were measured with the log-rank test and Cox proportional hazards model. Since patients diagnosed in 2016 could only be followed for up to 36 months, all survival curves were restricted to time intervals of 36 months. A sub-analysis by stage was also conducted, since 36 months may not have been a sufficient follow-up time to observe survival disparities for earlier stages of prostate cancer. For insurance status, hazard ratios were adjusted for age group (< 55, 50–70, > 70), race, PSA group (≤ 10, 10–20, > 20), biopsy Gleason score (≤ 6, 7, ≥ 8), and treatment with prostatectomy and/or radiotherapy or no local therapy. Changes in the distribution of insurance status were analyzed with the Pearson chi-square test, as changes in PCSS may be partially attributable to changes in the distribution of insurance status. Temporal changes in the distribution of patient characteristics between insurance groups were analyzed with a multinomial logistic regression using insured patients as the reference group to determine whether differences in patient characteristics from the pre- to post-USPSTF era were different between insured and Medicaid patients as well as between insured and uninsured patients. All statistical analyses were conducted using Stata/SE 15.0 (College Station, TX).

## Results

### In the post-USPSTF era, patients with prostate cancer presented with more advanced disease

Our study cohort was composed of 282,266 patients at least 40-years-old diagnosed with prostate cancer between January 2010 to December 2012 (pre-USPSTF) and January 2014 to December 2016 (post-USPSTF). There were 152,416 (54.00%) patients in the pre-USPSTF era and 129,850 (46.00%) patients in the post-USPSTF era. The median age of patients in the pre-USPSTF era was 65-years-old (IQR: 59–72-years-old), while that of patients in the post-USPSTF era was 66 (IQR: 60–72-years-old). Median follow-up time was 36 months (IQR: 36–36 months) in the pre-USPSTF era and 16 months (IQR: 8–26 months) in the post-USPSTF era (Table 1).

**Table 1:** Patient characteristics among insured and uninsured patients

	Insured		Medicaid			Uninsured		
	Pre-USPSTF (2010–2012) No. (% or IQR)	Post-USPSTF (2014–2016) No. (% or IQR)	Pre-USPSTF (2010–2012) No. (% or IQR)	Post-USPSTF (2014–2016) No. (% or IQR)	P-value^a^	Pre-USPSTF (2010–2012) No. (% or IQR)	Post-USPSTF (2014–2016) No. (% or IQR)	P-value^a^
Sample size	124,577 (54.88)	102,420 (45.12)	7,351 (47.63)	8,084 (52.37)		2,552 (63.15)	1,489 (36.85)	
Median age	65 (59–71)	66 (60–72)	66 (59–73)	63 (58–70)		61 (56–64)	62 (57–67)	
PSA, ng/mL					< 0.001			0.048
≤ 10	83,975 (75.52)	62,920 (68.69)	3,285 (53.02)	3,441 (49.21)		1,250 (55.07)	651 (49.28)	
10 < PSA ≤ 20	15,253 (13.72)	14,882 (16.25)	1,224 (19.75)	1,362 (19.48)		399 (17.58)	213 (16.12)	
> 20	11,969 (10.76)	13,799 (15.06)	1,687 (27.23)	2,189 (31.31)		621 (27.36)	457 (34.60)	
Total	111,197 (100.00)	91,601 (100.00)	6,196 (100.00)	6,992 (100.00)		2,270 (100.00)	1,321 (100.00)	
Biopsy Gleason Score					< 0.001			0.004
≤ 6	51,845 (45.40)	32,133 (34.23)	2,344 (37.41)	1,966 (28.60)		823 (36.45)	399 (31.10)	
7	43,401 (38.00)	39,845 (42.45)	2,364 (37.73)	2,893 (42.09)		865 (38.31)	491 (38.27)	
≥ 8	18,959 (16.60)	21,884 (23.32)	1,557 (24.85)	2,015 (29.31)		570 (25.24)	393 (30.63)	
Total	114,205 (100.00)	93,862 (100.00)	6,265 (100.00)	6,874 (100.00)		2,258 (100.00)	1,283 (100.00)	
Stage					0.002			< 0.001
Localized	99,138 (81.27)	75,388 (75.31)	5,153 (73.61)	5,199 (66.87)		1,762 (71.95)	987 (69.02)	
Regional	17,031 (13.96)	16,855 (16.84)	952 (13.60)	1,230 (15.82)		350 (14.29)	186 (13.01)	
Distant	5,813 (4.77)	7,859 (7.85)	895 (12.79)	1,346 (17.31)		337 (13.76)	257 (17.97)	
Total	121,982 (100.00)	100,102 (100.00)	7,000 (100.00)	7,775 (100.00)		2,449 (100.00)	1,430 (100.00)	
Treatment					< 0.001			0.524
No local therapy	64,670 (51.91)	58,790 (57.40)	5,089 (69.23)	5,570 (68.90)		1,634 (64.03)	1,041 (69.91)	
Prostatectomy and/or radiotherapy	59,907 (48.09)	43,630 (42.60)	2,262 (30.77)	2,514 (31.10)		918 (35.97)	448 (30.09)	
Total	121,982 (100.00)	100,102 (100.00)	7,000 (100.00)	7,775 (100.00)		2,449 (100.00)	1,430 (100.00)	
Race					< 0.001			0.004
White	87,783 (71.64)	70,744 (70.03)	2,570 (35.47)	3,115 (38.92)		1,137 (45.15)	575 (39.36)	
Black	18,268 (14.91)	15,773 (15.61)	1,955 (26.98)	2,265 (28.30)		766 (30.42)	543 (37.17)	
Hispanic	10,459 (8.54)	9,146 (9.05)	1,791 (24.72)	1,841 (23.00)		492 (19.54)	263 (18.00)	
Asian/Pacific Islander	5,624 (4.59)	5,015 (4.96)	873 (12.05)	715 (8.93)		119 (4.73)	78 (5.34)	
American Indian/Alaska Native	396 (0.32)	335 (0.33)	56 (0.77)	67 (0.84)		4 (0.16)	2 (0.14)	
Total	122,530 (100.00)	101,013 (100.00)	7,245 (100.00)	8,003 (100.00)		2,518 (100.00)	1,461 (100.00)	

^a^Multinomial logistic regression with generalized logit function with insured group as reference group

There was a modest but statistically significant change in insurance status from the pre- to post-USPSTF recommendation eras (p < 0.001, chi-squared). Specifically, insured patients decreased from 92.64 to 91.45%, while uninsured patients decreased from 1.90% to 1.33%. Contrastingly, the percentage of Medicaid patients increased from 5.47% to 7.22%. Additionally, patients in the post-USPSTF era presented with higher PSA, biopsy Gleason score (bGS), and higher summary stage, features consistent with more advanced disease (PSA: p < 0.001, bGS: p < 0.001, stage: p < 0.001, chi-squared). These post-USPSTF patients were also less likely to be treated with prostatectomy or radiotherapy (p < 0.001, chi-squared). Among these patients who did not receive local therapy, the proportion of those with regional and distant cancer increased across the eras from 3.11 to 4.08% and 8.89 to 12.71%, respectively, while those with localized disease decreased from 88.00 to 83.20% (Table 2; p < 0.001, chi-squared).

**Table 2:** Stage distribution of patients who did not receive local therapy from the pre-USPSTF (2010–2012) to post-USPSTF eras (2014–2016)

Stage	Pre-USPSTF Era (2010–2012) No. (%)	Post-USPSTF Era (2014–2016) No. (%)
Localized	71,430 (88.00)	62,333 (83.20)
Regional	2,527 (3.11)	3,057 (4.08)
Distant	7,218 (8.89)	9,525 (12.71)

Chi-squared: p < 0.001

Factors associated with decreased PCSS included coverage with Medicaid, uninsured, and non-Hispanic Black, higher PSA, bGS, and stage, and not receiving local treatment. In contrast, being non-Hispanic Asian/Pacific Islander was associated with increased PCSS. When analyzing the two eras separately using an adjusted Cox proportional hazards model, however, there were no survival disparities between non-Hispanic White and Black patients (Tables 3 and 4).

**Table 3:** Cox proportional hazards analysis of factors associated with prostate cancer-cause specific survival in the pre-USPSTF era (2010–2012)

	Sample size no. (%)	Adjusted HR (95% CI)	p-value
Age			
< 55	16,408 (10.77)	1 (Referent)	
55–70	91,149 (59.80)	0.946 (0.814–1.101)	0.474
> 70	44,859 (29.43)	1.555 (1.334–1.811)	< 0.001
Insurance status			
Insured	124,577 (92.64)	1 (Referent)	
Medicaid	7,351 (5.47)	1.309 (1.157–1.481)	< 0.001
Uninsured	2,552 (1.90)	1.256 (1.037–1.520)	0.020
Race			
Non-Hispanic White	102,334 (69.09)	1 (Referent)	
Non-Hispanic Black	23,540 (15.89)	1.072 (0.971–1.183)	0.167
Hispanic	14,410 (9.73)	0.953 (0.838–1.084)	0.465
Non-Hispanic Asian/Pacific Islander	7,320 (4.94)	0.654 (0.542–0.789)	< 0.001
Non-Hispanic American Indian/Alaskan Native	505 (0.34)	0.982 (0.542–1.777)	0.951
PSA, ng/mL			
≤ 10	96,214 (74.06)	1 (Referent)	
10 < PSA ≤ 20	18,271 (14.06)	1.403 (1.221–1.613)	< 0.001
> 20	15,421 (11.87)	2.280 (2.017–2.578)	< 0.001
Biopsy Gleason Score			
≤ 6	63,638 (45.81)	1 (Referent)	
7	51,723 (37.23)	2.036 (1.690–2.452)	< 0.001
≥ 8	23,560 (16.96)	5.639 (4.701–6.765)	< 0.001
Stage			
Localized	119,995 (82.09)	1 (Referent)	
Regional	18,801 (12.86)	2.478 (2.123–2.892)	< 0.001
Distant	7,384 (5.05)	12.634 (11.286–14.143)	< 0.001
Treatment			
No local therapy	87,246 (57.24)	1 (Referent)	
Prostatectomy and/or radiotherapy	65,170 (42.76)	0.209 (0.176–0.250)	< 0.001

**Table 4:** Cox proportional hazards analysis of factors associated with prostate cancer-cause specific survival in the post-USPSTF era (2014–2016)

	Sample size no. (%)	Adjusted HR (95% CI)	p-value
Age			
< 55	11,843 (9.12)	1 (Referent)	
55–70	79,397 (61.15)	1.235 (0.961–1.587)	0.099
> 70	38,610 (29.73)	1.973 (1.533–2.540)	< 0.001
Insurance status			
Insured	102,420 (91.45)	1 (Referent)	
Medicaid	8,084 (7.22)	1.230 (1.040–1.454)	0.015
Uninsured	1,489 (1.33)	0.946 (0.642–1.394)	0.780
Race			
Non-Hispanic White	84,102 (67.28)	1 (Referent)	
Non-Hispanic Black	20,939 (16.75)	1.125 (0.974–1.298)	0.108
Hispanic	12,994 (10.39)	1.006 (0.838–1.208)	0.948
Non-Hispanic Asian/Pacific Islander	6,505 (5.20)	0.738 (0.571–0.954)	0.020
Non-Hispanic American Indian/Alaskan Native	463 (0.37)	1.371 (0.755–2.490)	0.300
PSA, ng/mL			
≤ 10	74,416 (67.56)	1 (Referent)	
10 < PSA ≤ 20	18,022 (16.36)	1.239 (1.010–1.519)	0.039
> 20	17,715 (16.08)	1.985 (1.672–2.358)	< 0.001
Biopsy gleason score			
≤ 6	41,749 (35.48)	1 (Referent)	
7	48,907 (41.56)	2.328 (1.648–3.288)	< 0.001
≥ 8	27,019 (22.96)	7.023 (5.024–9.815)	< 0.001
Stage			
Localized	94,477 (76.71)	1 (Referent)	
Regional	18,878 (15.33)	2.497 (1.994–3.127)	< 0.001
Distant	9,801 (7.96)	11.763 (9.963–13.887)	< 0.001
Treatment			
No local therapy	81,388 (62.68)	1 (Referent)	
Prostatectomy and/or radiotherapy	48,462 (37.32)	0.201 (0.154–0.263)	< 0.001

### Adjusted PCSS disparities between insured and uninsured groups disappeared in the post-USPSTF era

Across both eras, there were 226,997 insured, 15,435 Medicaid, and 4,041 uninsured patients. Of the insured patients, 54.88% (n = 124,577) and 45.12% (n = 102,420) were in the pre- and post-USPSTF eras, respectively. Of the Medicaid patients, 47.63% (n = 7,351) and 52.37% (n = 8,084) were in the pre- and post-eras, respectively. Of the uninsured patients, 63.15% (n = 2,552) and 36.85% (n = 1,489) were in the pre- and post-USPSTF eras, respectively.

From the pre- to post-USPSTF era, the changes in PSA between insured and uninsured patients as well as insured and Medicaid patients were statistically significant with insured patients experiencing a greater increase in those with a PSA greater than 10 ng/ml but less than or equal to 20 ng/ml than uninsured and Medicaid patients (p = 0.048 and p < 0.001, respectively, multinomial logistic regression with generalized logit function [LR]). Compared to insured patients, however, uninsured patients experienced a greater increase in those with a PSA greater than 20 ng/ml. Insured patients also presented with higher bGS than uninsured and Medicaid patients with the proportion of those with bGS less than or equal to 6 decreasing from 45.40% (n = 51,845) to 34.23% (n = 32,133), bGS of 7 increasing from 38.00% (n = 43,401) to 42.45% (n = 39,845), and bGS greater than or equal to 8 increasing from 16.60% (n = 18,959) to 23.32% (n = 21,884) (p = 0.004 and p < 0.001, respectively, LR). Lastly, while insured and uninsured patients experienced similar shifts in local treatment from the pre- to post-USPSTF era (p = 0.524, LR), insured patients experienced a greater decrease in prostatectomy and radiotherapy relative to Medicaid patients (p < 0.001, LR).

**Fig. 1:**
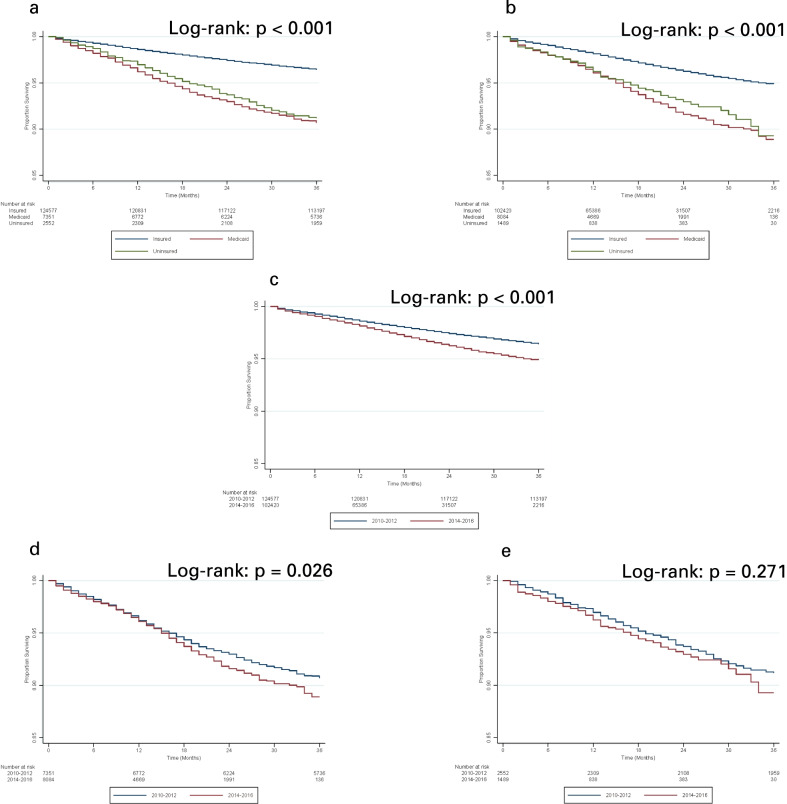
**A** 3-year prostate cancer-specific survival of insured and uninsured patients with prostate cancer in 2010–2012 (pre-USPSTF era). **B** 3-year prostate cancer-specific survival of insured and uninsured patients with prostate cancer in 2014–2016 (post-USPSTF era) **C** 3-year prostate cancer-specific survival of insured patients with prostate cancer in 2010–2012 (pre-USPSTF era) and 2014–2016 (post-USPSTF era). **D** 3-year prostate cancer-specific survival of Medicaid patients with prostate cancer in 2010–2012 (pre-USPSTF era) and 2014–2016 (post-USPSTF era). **E** 3-year prostate cancer-specific survival of uninsured patients with prostate cancer in 2010–2012 (pre-USPSTF era) and 2014–2016 (post-USPSTF era). Prostate cancer-specific survival of insured and Medicaid patients with prostate cancer worsened from 2010–2012 to 2014–2016, while that of uninsured patients did not change

During the pre-USPSTF era, uninsured patients experienced worse PCSS than insured patients (Fig. 1a; HR 2.512, 95% CI 2.813–2.889, p < 0.001). This survival disparity narrowed as a result of a statistically significant decrease in PCSS among insured patients (Figs. 1b and 1c; p < 0.001, log-rank) and no statistically significant change in PCSS among uninsured patients (Fig. 1e; p = 0.271, log-rank) (HR 1.980, 95% CI 1.564–2.505, p < 0.001). When adjusted for factors such as age, race, and PSA, however, the PCSS disparity between insured and uninsured patients in the pre-USPSTF era disappeared altogether in the post era (Tables 3 and 4; pre-USPSTF: aHR 1.256, 95% CI 1.037–1.520, p = 0.020; post-USPSTF: aHR 0.946, 95% CI 0.642–1.394, p = 0.780). In addition, in both the pre- and post-USPSTF era, there was no survival disparity between White and Blacks when insurance status was adjusted in addition to clinical features (Table 3, aHR 1.072, 95% CI 0.971–1.183, p = 0.167). In contrast, the survival disparity between Medicaid and insured patients from the pre- to post-USPSTF era did not significantly change with Medicaid patients continuing to experience decreased survival relative to insured patients (Fig. 1d; pre: aHR 1.309, 95% CI 1.157–1.481, p < 0.001; post: aHR 1.230, 95% CI 1.040–1.454, p = 0.015; p = 0.553, two-tailed test).

### While insured patients experienced worse survival, and uninsured patients did not observe a survival change across all stages of prostate cancer, adjusted PCSS disparities between insured and uninsured groups disappeared in the post-USPSTF era for distant prostate cancer

**Fig. 2:**
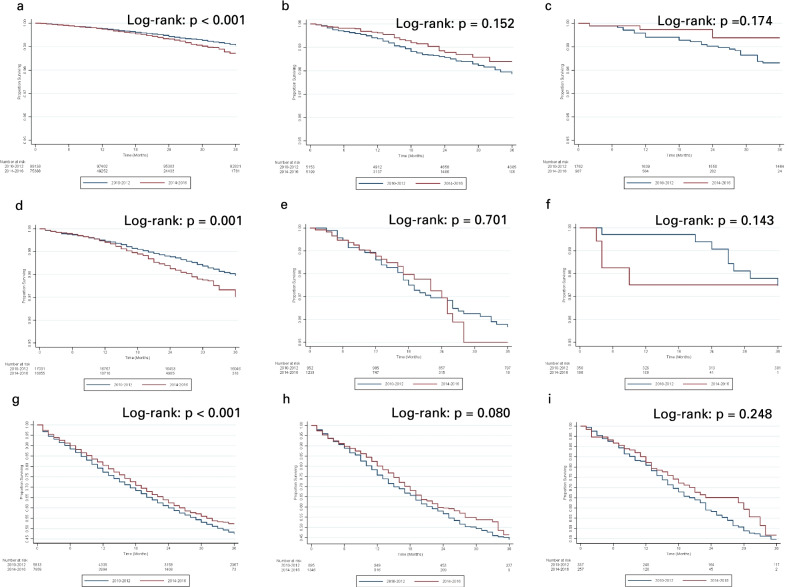
**A** 3-year prostate cancer-specific survival of insured patients with localized prostate cancer in 2010–2012 (pre-USPSTF era) and 2014–2016 (post-USPSTF era). **B** 3-year prostate cancer-specific survival of Medicaid patients with localized prostate cancer in 2010–2012 (pre-USPSTF era) and 2014–2016 (post-USPSTF era). **C** 3-year prostate cancer-specific survival of uninsured patients with localized prostate cancer in 2010–2012 (pre-USPSTF era) and 2014–2016 (post-USPSTF era). **D** 3-year prostate cancer-specific survival of insured patients with regional prostate cancer in 2010–2012 (pre-USPSTF era) and 2014–2016 (post-USPSTF era). **E** 3-year prostate cancer-specific survival of Medicaid patients with regional prostate cancer in 2010–2012 (pre-USPSTF era) and 2014–2016 (post-USPSTF era). **F** 3-year prostate cancer-specific survival of uninsured patients with regional prostate cancer in 2010–2012 (pre-USPSTF era) and 2014–2016 (post-USPSTF era). **G** 3-year prostate cancer-specific survival of insured patients with metastatic prostate cancer in 2010–2012 (pre-USPSTF era) and 2014–2016 (post-USPSTF era). **H** 3-year prostate cancer-specific survival of Medicaid patients with metastatic prostate cancer in 2010–2012 (pre-USPSTF era) and 2014–2016 (post-USPSTF era). **I** 3-year prostate cancer-specific survival of uninsured patients with metastatic prostate cancer in 2010–2012 (pre-USPSTF era) and 2014–2016 (post-USPSTF era). Prostate cancer-specific survival of insured patients across all stages of prostate cancer worsened from 2010–2012 to 2014–2016, while Medicaid and uninsured patients across all stages did not change

Insured patients across all stages of prostate cancer experienced a statistically significant decrease in survival from the pre- to post-USPSTF eras, while Medicaid and uninsured patients did not experience a survival change (Fig. 2). In an adjusted Cox proportional hazards model stratified by stage, however, there were no statistically significant survival disparities between insured and uninsured patients with localized or regional prostate cancer in both the pre- and post-USPSTF eras (Table 5; pre-USPSTF, uninsured and localized: aHR 1.089, 95% CI 0.657–1.803, p = 0.742; post-USPSTF, uninsured and localized: aHR 0.805, 95% CI 0.256–2.531, p = 0.711; pre-USPSTF uninsured and regional: aHR 1.556, 95% CI 0.758–3.191, p = 0.228; post-USPSTF uninsured and regional: subgroup too small for hazard ratio calculation). For patients with distant prostate cancer, there was a survival disparity between insured and uninsured patients in the pre-USPSTF era that disappeared in the post-USPSTF era (pre-USPSTF, uninsured and distant: aHR 1.244, 95% CI 1.002–1.545, p = 0.048; post-USPSTF, uninsured and distant: aHR 1.048, 95% CI 0.693–1.586, p = 0.824).

**Table 5:** Cox proportional hazards analysis of insurance status associated with prostate cancer-cause specific survival by era and stage

	Sample size no. (%)	Adjusted HR (95% CI)	p-value
*Pre-USPSTF Era (2010–2012)*			
Insurance status for localized PCa			
Insured	99,138 (93.48)	1 (Referent)	
Medicaid	5,153 (4.86)	1.373 (1.060–1.778)	0.016
Uninsured	1,762 (1.66)	1.089 (0.657–1.803)	0.742
Insurance status for regional PCa			
Insured	17,031 (92.90)	1 (Referent)	
Medicaid	952 (5.19)	1.474 (0.985–2.207)	0.059
Uninsured	350 (1.91)	1.556 (0.758–3.191)	0.228
Insurance status for distant PCa			
Insured	5,813 (82.51)	1 (Referent)	
Medicaid	895 (12.70)	1.254 (1.080–1.457)	0.003
Uninsured	337 (4.78)	1.244 (1.002–1.545)	0.048
*Post-USPSTF Era (2014–2016)*			
Insurance status for localized PCa			
Insured	75,388 (92.42)	1 (Referent)	
Medicaid	5,199 (6.37)	0.999 (0.633–1.575)	0.995
Uninsured	987 (1.21)	0.805 (0.256–2.531)	0.711
Insurance status for regional PCa			
Insured	16,855 (92.25)	1 (Referent)	
Medicaid	1,230 (6.73)	1.662 (1.000–2.761)	0.050
Uninsured	186 (1.02)	N/A	N/A
Insurance status for distant PCa			
Insured	7,859 (83.06)	1 (Referent)	
Medicaid	1,346 (14.23)	1.242 (1.023–1.506)	0.028
Uninsured	257 (2.72)	1.048 (0.693–1.586)	0.824

## Discussion

In the present study, we show that patients in the post-USPSTF era presented with more adverse clinicopathologic features, such as higher PSA, bGS, and stage. In addition, the disparity in prostate cancer survival between the insured and uninsured disappeared in the post-USPSTF era due to the worse outcome in the insured group. The decrease in PCSS of insured patients can largely be explained by the decrease in PCSS of insured patients with low PSA. Importantly, we found that the black race was not a predictor of worse prostate cancer survival outcome when insurance status was adjusted.

Our observation of worse prostate cancer outcome following the 2012 USPSTF's recommendation against PSA-based screening is consistent with that of previous studies, which report that the USPSTF’s 2012 PSA screening recommendation has had profound effects on the presentation and management of prostate cancer. Our previous work found that prostate-cancer specific survival for all prostate cancer patients worsened after 2012 [10]. A separate study found that prostate cancer screening rates decreased from 31.8% in 2008 to 24.2% in 2013, while another reported a 16.4% decrease in clinical workup for men with high PSA [11, 12]. As a result of decreased screening, studies indicate that primary care providers are referring their patients for urologic evaluation at higher PSA values [13]. Moreover, Dalela et al. described a 4.2% increase in men with Gleason 8 prostate cancer from 2011 to 2013 [14]. Altogether, studies have predicted that forgoing PSA screening would result in a 13–20% increase in death from prostate cancer and twofold increase in the incidence of metastatic disease [15, 16]. Patients in the post-USPSTF era were also less likely to receive local therapy, possibly due to a higher proportion of those with regional and distant disease and increased number of deaths. This finding is also consistent with studies that have reported increased adoption of active surveillance during this time period, meaning that patients with localized disease in the post-USPSTF era may have been more likely to delay the need for treatment [17]. Furthermore, in 2012, the results of the Prostate Cancer Intervention versus Observation Trial (PIVOT) study were published, which demonstrated that radical prostatectomy did not significantly improve overall or prostate cancer-specific survival among patients with localized prostate cancer [18]. Thus, providers and patients may have been more inclined to delay local therapy in the post-USPSTF era. Additionally, while considered statistically significant, the decrease in the number of insured patients from the pre- to post-USPSTF era was very small at 1.19%.

The disappearance of PCSS disparity between insured and uninsured patients after the USPSTF’s 2012 screening recommendation occurred due to a decrease in PCSS among insured patients combined with no change in PCSS among uninsured patients. This finding suggests that in the pre-USPSTF era, insured men were more likely to be screened for prostate cancer than uninsured men, likely due to more consistent urologic care as well as insurance coverage of PSA screening. Aghdam and colleagues reported that insurance status was the most protective factor against presenting with metastatic prostate cancer [19]. Kearns et al*.*, however, found that among privately insured patients aged 40–64, the rate of PSA testing decreased from 27.6% in 2010–2012 to 25.9% in 2014, suggesting that the recommendation may have contributed to a decrease in PSA screening rates among insured patients [20]. Thus, in recommending against PSA-based screening for prostate cancer, the USPSTF may have discouraged more insured patients from being screened for prostate cancer, while uninsured patients were just as likely to remain unscreened. While insured patients are more likely to have a primary care provider (PCP), studies report that the USPSTF’s screening recommendation led to a 39% decrease in PSA testing by PCPs along with 62.3% of PCPs being less likely to screen or cease screening altogether, thereby partially negating the protective effect of having a PCP in managing prostate cancer [21–24]. Additionally, based on the National Health Interview Survey (NHIS), the overall prevalence of PSA screening rate was 35.1% in 2010 before the recommendation. After the recommendation, it decreased to 30.1% in 2015 [25]. Collectively, we propose that by discouraging PSA-based prostate cancer screening, insured patients are essentially behaving similar to uninsured patients with respect to prostate cancer screening. These findings support the idea that practicing clinicians should appropriately encourage patients to get screened for prostate cancer regardless of insurance status.

In contrast to the survival disparity between insured and uninsured patients that disappeared from the pre- to post-USPSTF era, that between insured and Medicaid patients did not significantly change, suggesting that the decrease in PCSS among Medicaid patients approximated that of insured patients. Moreover, Medicaid patients consistently experienced worse PCSS than insured patients across both eras and even uninsured patients in the post-USPSTF era. Previous studies have found that compared to insured patients, Medicaid patients with prostate cancer are more likely to present with metastatic disease and have higher prostate cancer-specific mortality [26]. Suh and colleagues also reported that Medicaid patients with prostate cancer may have a higher risk of death than uninsured patients with prostate cancer [27].

In the sub-analysis stratified by stage, the only survival disparities between insured and uninsured patients in the pre-USPSTF era were observed in those with distant prostate cancer. This finding may be explained by the follow-up period of 36 months, as this timeframe may not be long enough to observe survival disparities among patients with localized and regional prostate cancer given that the 5-year survival of these patients has been reported to be up to more than 99%. In contrast, given the 5-year survival rate of 31% for distant disease, a 36-month follow up time was likely sufficient to observe changes in survival disparities for those with distant prostate cancer [28]. Thus, given our observation that insurance survival disparities disappeared from the pre- to post-USPSTF era in the overall cohort, it is possible that a longer follow-up period for patients with localized and regional prostate cancer would reveal a similar trend to that of distant prostate cancer patients.

Previously, we have reported a survival disparity between Whites and Blacks in the pre-USPSTF era [10]. While this aforementioned study did not adjust for socioeconomic factors, the present investigation adjusted for insurance status and found that the disparity in prostate cancer survival between Whites and Blacks disappeared. Such a result is consistent with the body of published data that has demonstrated that socioeconomic factors are major reasons for the survival disparity between Whites and Blacks. For example, after adjusting for clinical and nonclinical factors related to access to care, Wen and colleagues reported that the Black-White survival disparity significantly decreased from 51 to 20% higher mortality among Black patients. Moreover, insurance status was one of the most important nonclinical factors that contributed to this racial disparity [29]. A separate study found that after adjusting for poverty, income, and a composite socioeconomic variable, the risk of mortality in African-American men with prostate cancer was no longer significantly different from that of Whites [30]. Based on these observations, we support the view that engaging the Black community and addressing its various socioeconomic disadvantages will be necessary to significantly reduce the prostate cancer survival disparity between Whites and Blacks.

The main strength of our study was the use of a large population-based database, which enabled the analysis of real-world trends and outcomes. Several limitations of our study include those inherent to the SEER database including the lack of screening data on patients with prostate cancer in SEER, the increased censoring of patients in the post-USPSTF era compared to the pre-USPSTF era, the assumption that insurance coverage of patients has remained consistent over time, the short follow-up period of 3 years, and the lack of SEER data on actual prostate cancer screening rates. Additionally, while outcomes such as biochemical-recurrence-free survival or metastasis-free survival may have been more appropriate, the SEER database does not include these outcomes, making PCSS the best available outcome. Using PCSS as the primary outcome and a follow-up period of only 3 years may have resulted in the number of patient deaths being too small to detect a survival disparity in the post-recommendation era. However, we did apply the same primary outcome and follow-up period for patients in the pre-USPSTF era and still found a survival disparity, indicating that a factor other than the small number of observed patient deaths is responsible for the disappearance of a survival disparity between insured and uninsured patients. Regardless, a difference in PSA screening rates is not necessarily the only change that occurred between eras. Thus, this study should be considered a hypothesis-generating study from which future studies may test these hypotheses on additional databases.

## Conclusion

The USPSTF’s 2012 PSA screening recommendation may have had unintended, detrimental effects on survival disparities based on insurance status, suggesting that alternative approaches to screening may be necessary for improved survival among both insured and uninsured patients.

## Data Availability

This study used publicly available data from the Surveillance, Epidemiology, and End Results Program (SEER) database at https://seer.cancer.gov/data/.

## Abbreviations

PSA: Prostate-specific antigen
ERSPC: European Randomized Study of Screening for Prostate Cancer
PLCO: United States Prostate, Lung, Colorectal, and Ovarian Cancer Screening Trial
SPCG-4: Scandinavian Prostate Cancer Group Study Number Four
PIVOT: Prostate Cancer Intervention Versus Observation Trial
USPSTF: United States Preventive Services Taskforce
AUA: American Urologic Association
SEER: Surveillance, Epidemiology, and End Results
bGS: Biopsy Gleason score
PCSS: Prostate cancer-specific survival
PCP: Primary care provider
